# AGESMind clinical trial: SocialMIND® results at 16 weeks

**DOI:** 10.1192/j.eurpsy.2023.1028

**Published:** 2023-07-19

**Authors:** M. P. Vidal-Villegas, A. Abad Pérez, P. Herrero Ortega, A. Oliva Lozano, J. Garde González, J. Andreo-Jover, A. Muñoz-Sanjosé, R. Mediavilla, B. Rodríguez-Vega, G. Lahera, Á. Palao-Tarrero, C. Bayón-Pérez, M. F. Bravo-Ortiz

**Affiliations:** 1 Hospital La Paz Institute for Health Research (IdiPAZ), Madrid, Spain; 2 Department of Psychiatry, Autonomous University of Madrid (UAM); 3 Department of Psychiatry, Clinical Psychology and Mental Health, La Paz University Hospital; 4 Centro de Investigación Biomédica en Red de Salud Mental (CIBERSAM); 5 Principe de Asturias University Hospital, University of Alcala de Henares, Madrid, Spain

## Abstract

**Introduction:**

Early intervention on a first psychotic episode is fundamental for a more favorable prognosis, and it usually combines pharmacological treatment, which mainly affects positive psychotic symptoms, with interventions that can improve the rest of the symptoms and associated problems such as deterioration in social functioning (Harvey & Penn, 2010; Fusar-Poli, McGorry & Kane, 2017). While Mindfulness is gaining more and more prominence in the field of psychotherapy (Chan et al., 2019; Cillesen et al., 2019), social cognition and social functioning are being researched as key targets on which to intervene after a first psychotic episode (Green, Horan & Lee, 2015).

SocialMIND® is a mindfulness-based social cognition training tailor-made to improve social functioning in people who have suffered a first psychotic episode within the last five years. It is currently being compared with a group Psychoeducational Multicomponent Intervention (PMI) in a Randomized Controlled Trial (RCT) (Mediavilla et al., 2019). Both group psychotherapies include 17 sessions delivered over a 9 month period: 8 weekly sessions, 4 biweekly sessions and 5 monthly sessions.

The results of SocialMIND® at 8 weeks showed improvements in social cognition and social functioning, specifically on affective social cognition and self-care (Mediavilla et al., 2021).

**Objectives:**

To evaluate the efficacy of SocialMIND® in improving social functioning, measured by the Personal and Social Functioning (PSP) scale 16 weeks after starting the intervention, in people who have suffered a first psychotic episode in the last 5 years.

**Methods:**

Randomized, controlled pilot trial (use of a psychoeducational multicomponent intervention or PMI as active comparator) of two parallel groups (SocialMIND® and PMI) with a 1:1 ratio using a blind evaluator.

**Results:**

No statistically significant differences were found in the social functioning variable between the two treatment arms. Intragroup differences are observed in other secondary variables studied (social cognition) 16 weeks after starting the interventions.

**Conclusions:**

SocialMIND® has not been shown to be more effective than a PMI in improving social functioning at 16 weeks after starting the intervention in people who have suffered a first psychotic episode in the five years prior to being included in the study.

**Disclosure of Interest:**

None Declared

